# Reviews

**Published:** 1882-04

**Authors:** 


### Reviews.

---

**Home and Climatic Treatment of Pulmonary Consumption on the Basis of Modern Doctrines**. By J. Hilgard Tyndale, M. D. New York: Bermingham & Co., Publishers, 1260 and 1262 Broadway. Price 50 cents. 1882.

This little volume of 174 pages treats of the important disease, commonly called consumption, under five heads: Pathology in Consumption; A Treatment in General; Home Treatment; Climatic Treatment; General Conclusions. After carefully examining its contents we fail to find anything new or original in the author's pathology and treatment. The work is commended to our readers as one of many issued by writers, more ambitious for notoriety than to serve the profession with fresh and original ideas in pathology and therapeutics.

---

**A Manual of Organic Materia Medica, Being a Guide to Materia Medica of the Vegetable and Animal Kingdoms for the use of Students, Druggists, Pharmacists and Physicians.** By John M. Maisch, Phar. D., Prof. of Mat. Med. and Botany in the Philadelphia College of Pharmacy. With many illustrations. Philadelphia: H. C. Lea's Sons & Co. Price $2.75.

This is a good book for pharmacists for whom it is more especially designed. The medical properties and doses of the various drugs are simply stated, but there is no attempt to give instructions on their therapeutical applications. It is well illustrated and handsomely printed. Prof. Maisch is a master in his department, and the work is an evidence of true progress in pharmacology.

